# An absolute approach to using whole exome DNA and RNA workflow for cancer biomarker testing

**DOI:** 10.3389/fonc.2023.1002792

**Published:** 2023-03-13

**Authors:** Richa Malhotra, Vyomesh Javle, Nishtha Tanwar, Pooja Gowda, Linu Varghese, Anju K, Nandhitha Madhusudhan, Nupur Jaiswal, Bhargavi K. S., Manjima Chatterjee, Kumar Prabhash, Peddagangannagari Sreekanthreddy, Kshitij D. Rishi, Hitesh M. Goswami, Vidya H. Veldore

**Affiliations:** ^1^ 4baseCare Onco Solutions Pvt. Ltd., Bangalore, India; ^2^ Department of Medical Oncology, Tata Memorial Centre, Mumbai, India

**Keywords:** comprehensive genomic profiling (CGP), microsatellite instability (MSI), RNA exome, tumor mutational burden (TMB), whole exome sequencing (WES)

## Abstract

**Introduction:**

The concept of personalized medicine in cancer has emerged rapidly with the advancement of genome sequencing and the identification of clinically relevant variants that contribute to disease prognosis and facilitates targeted therapy options. In this study, we propose to validate a whole exome-based tumor molecular profiling for DNA and RNA from formalin-fixed paraffin-embedded (FFPE) tumor tissue.

**Methods:**

The study included 166 patients across 17 different cancer types. The scope of this study includes the identification of single-nucleotide variants (SNVs), insertions/deletions (INDELS), copy number alterations (CNAs), gene fusions, tumor mutational burden (TMB), and microsatellite instability (MSI). The assay yielded a mean read depth of 200×, with >80% of on-target reads and a mean uniformity of >90%. Clinical maturation of whole exome sequencing (WES) (DNA and RNA)- based assay was achieved by analytical and clinical validations for all the types of genomic alterations in multiple cancers. We here demonstrate a limit of detection (LOD) of 5% for SNVs and 10% for INDELS with 97.5% specificity, 100% sensitivity, and 100% reproducibility.

**Results:**

The results were >98% concordant with other orthogonal techniques and appeared to be more robust and comprehensive in detecting all the clinically relevant alterations. Our study demonstrates the clinical utility of the exome-based approach of comprehensive genomic profiling (CGP) for cancer patients at diagnosis and disease progression.

**Discussion:**

The assay provides a consolidated picture of tumor heterogeneity and prognostic and predictive biomarkers, thus helping in precision oncology practice. The primary intended use of WES (DNA+RNA) assay would be for patients with rare cancers as well as for patients with unknown primary tumors, and this category constitutes nearly 20–30% of all cancers. The WES approach may also help us understand the clonal evolution during disease progression to precisely plan the treatment in advanced stage disease.

## 1 Introduction

Rapid advancements in sequencing technology and its easy access have brought down the cost of genome sequencing significantly in the last two decades, and hence, genomic or personalized medicine has become an integral part of cancer management.

**Figure 1:**
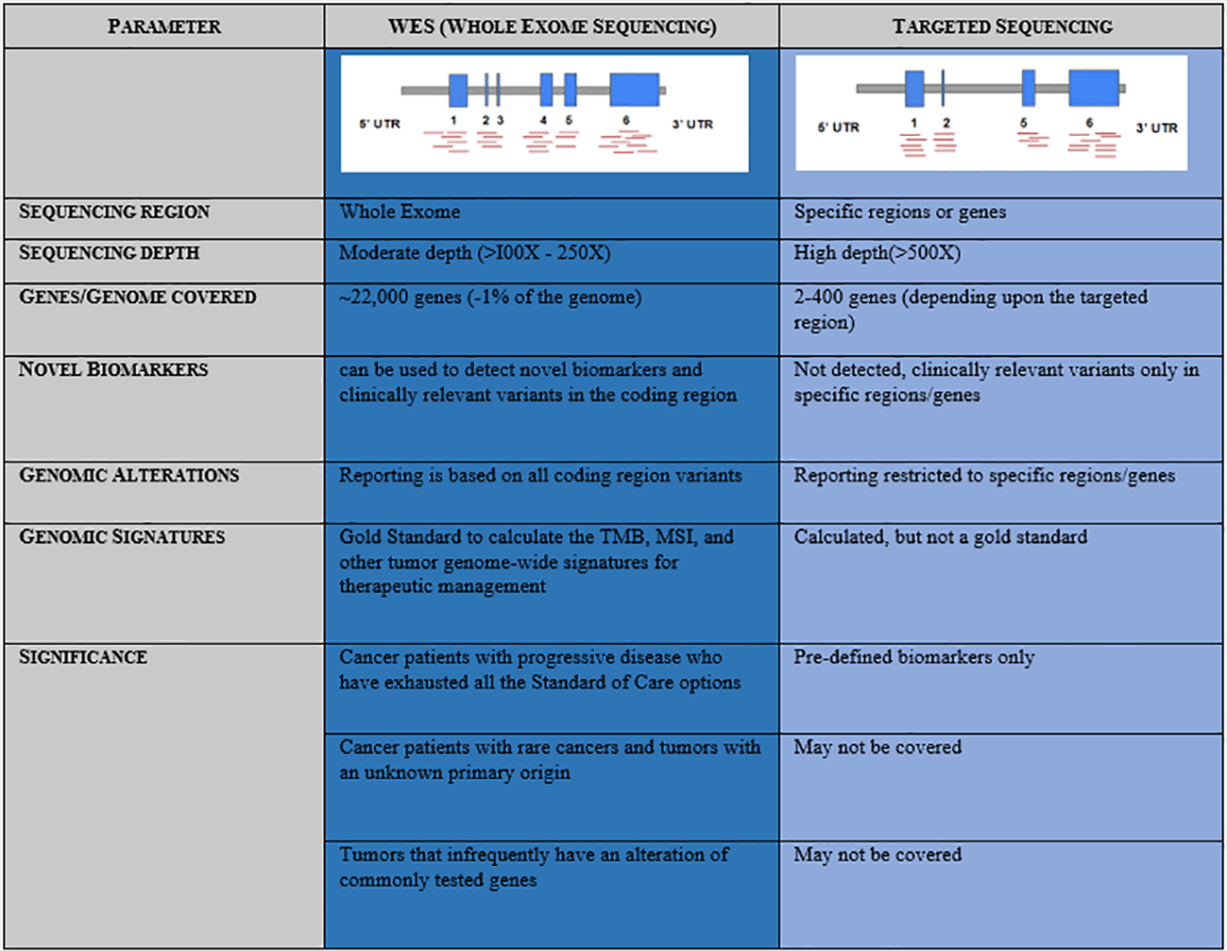
Comparative representation and advantage of Whole Exome Sequencing (WES) over Targeted Sequencing panels.

A precise, comprehensive diagnostic workup in cancer requires advanced high-throughput technologies beyond microscopy, immunohistochemistry, and single gene tests to understand the disease complexity, particularly when the world is witnessing a rapid increase in the onset of cancers with increased mortality (GLOBOCAN 2020) (1). In recent clinical practice, next-generation sequencing (NGS) has emerged as a powerful approach that provides a holistic picture of the disease. Targeted NGS panels identify mutations in a limited number of genes and could miss a few rare yet important driver mutations, which are associated with the mechanism of resistance or aggressive disease biology (2). In contrast, whole exome sequencing (WES) has gained much more importance in large-scale population-based clinical research studies, thus remains an advanced solution to detect cancer predisposition, mutations associated with disease onset, and progression that facilitates access to the standard of care as well as novel targeted therapies (3, 4). Mutational signatures, tumor mutational burden (TMB), and microsatellite instability (MSI) are best uncovered using the whole exome-based NGS panels, as it provides the advantage of a complete footprint of the coding region (**Figure 1**) (5, 6). However, it is less clear that WES is advantageous over a targeted panel for MSI detection. MSI being a marker with different loci mutated in different cancers, broader gene panels are important. Based on the evidence from different clinical studies, it is very clear that NGS-based MSI detection requires cross-validation with standard clinical practices such as MMR-IHC and MSI-PCR demonstrating 100% accuracy (7–14). Taken together, the WES panel for tumor DNA and RNA helps to facilitate tailor-made treatment decisions in clinical practice in the most comprehensive manner.

We present here a clinical validation of a whole exome (DNA and RNA) comprehensive NGS panel for tumor specimens [formalin-fixed paraffin-embedded (FFPE)] and its application in clinics with a reasonable turnaround time of 21 working days supporting physicians and cancer patients. The scope of the testing includes identifying single-nucleotide variants (SNVs), insertions/deletions (INDELS), copy number alterations (CNAs), gene fusions, TMB, and MSI. We believe that such a comprehensive approach could simplify the diagnostic algorithm, replacing the gene-specific or panel-based testing, and helps in better stratification of patients and their treatment management. The major application of this approach at the time of diagnosis may include tumors with rare histology as well as tumors where the primary origin could not be deciphered.

## 2 Materials and methods

### 2.1 Patient cohort for assay validation

**Figure 2:**
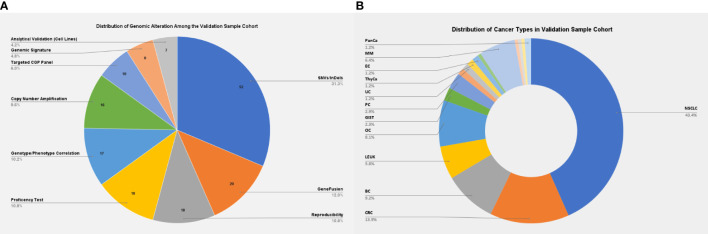
**(A)** Distribution of genomic alterations among the validation sample cohort. **(B)** Distribution of cancer types among the validation sample cohort. NSCLC, non-small cell lung carcinoma; CRC, colorectal cancer; BC, breast cancer; LEUK, leukemia; OC, ovarian cancer; GIST, gastrointestinal stromal tumors; PC, prostatic cancer; EC, endometrial cancer; MM, multiple myeloma; PanCa, pancreatic cancer.

The present validation cohort constitutes a total of 166 samples across 17 cancer types, which includes: 1) a comprehensive set of representative clinical samples (N = 129) and 2) pre-characterized cancer cell lines, reference standards from Horizon Discovery (traceable from the National Institute of Standards and Technology), and proficiency testing material (N = 37) (**Figure 2**). The study was conducted according to the principles of the Declaration of Helsinki and as per the International Council for Harmonisation of Technical Requirements for Pharmaceuticals for Human Use (ICH) and Good Clinical Practice (GCP) guidelines (15, 16). Written informed consent was obtained from the enrolled patients for the use of de-identified data for research publications. The study was approved by an independent ethics committee and review board (Jehangir Clinical Development Center JCDC, India).

### 2.2 Sample selection, library preparation, and sequencing

Three different sample types were used, that includes FFPE DNA, Genomic DNA derived from blood and FFPE RNA. For the analytical validation, FFPE DNA were sourced from Horizon Discovery. The external quality assurance was performed using DNA obtained as part of proficiency testing.

The germline DNA was isolated from peripheral blood using QIAamp DNA Blood Kit (Cat. No. 51104). 200 ng of genomic DNA was used for Whole Exome library preparation using the standard procedures as per the manufacturer's instructions using the Agilent DNA Prep with Enrichment kit (Cat. No. 5191-6874).

The FFPE blocks with minimum tumor surface area ≥ 5 mm^2^ and tumor content ≥ 10% (i.e., roughly 150 viable tumor cells per high- power field (HPF) on microscopy as per histological examination) were processed for tumor genomic DNA and RNA extraction by using All Prep FFPE DNA/RNA kit Cat. No. 80234 (17) (Qiagen, Valencia, CA, USA). Quality control (QC)-qualified DNA/RNA samples were processed for library preparation, which includes fragmentation, adapter addition, amplification, and capturing of exonic regions of genomic DNA through overnight hybridization of exon-specific probes. Agilent DNA/RNA Prep with Enrichment kit (Cat. No. 5191-6874) (18) was used to prepare the DNA-exome and RNA-Seq (RNA-exome) libraries. The prepared libraries underwent QC analysis for the detection of library fragment size and concentration. A qualified NGS library had at least 10-nM concentration with a single distinct peak of approximately 300 bp (**Figure S1**). The qualified NGS libraries were subjected to paired-end (2 × 150 read length configuration) sequencing on the NextSeq™ Systems (Illumina Inc., San Diego, CA, USA) at a median coverage of 200X (**Table S1**).

The quality of the DNA and the NGS libraries are verified by Qubit and Bio-analyzer. **Figure S1** provides the information that the QCs are measured using the Bio-analyzer rather than the standard spectrophotometric methods.

### 2.3 Bioinformatics analysis for tumor genomic alterations

**Figure 3:**
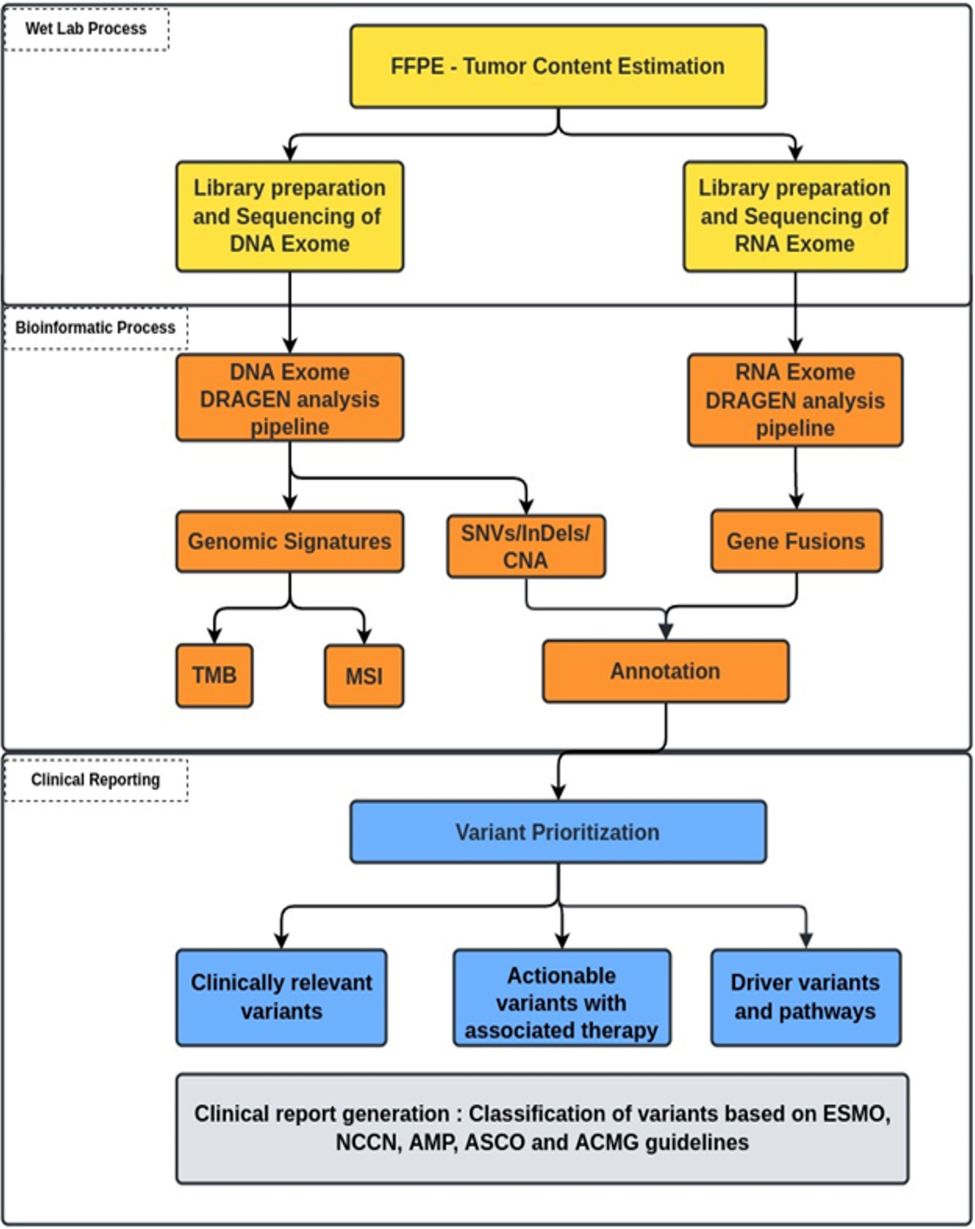
Schematic workflow from sample to clinical report.

Raw sequencing reads from high-throughput sequencers were obtained in fastq formats. The reads were further analyzed using a customized bioinformatics pipeline (**Figure 3**) to detect genomic alterations (SNVs, INDELS, CNAs, and gene fusions). Illumina DRAGEN Somatic Pipeline (Illumina DRAGEN Bio-IT^TM^ Platform v3.6) and Illumina DRAGEN RNA Pipeline (Illumina DRAGEN Bio-IT^TM^ Platform v3.6) were used to analyze exome data for DNA and RNA, respectively. **Figures S2A, B** describe the workflow. High-quality short-read sequences were aligned to human reference sequence version hg19/GRCh37 using DRAGEN-aligner followed by flagging off the duplicate sequencing reads. The SNVs, INDELS, and CNAs (>6) were detected using DNA exome data, while gene fusions were detected using RNA exome data.

### 2.4 Variant annotations and databases

Variants were annotated using an in-house developed pipeline with modules of population databases (1000 Genomes and ExAC), *in silico* gene prioritization tools (CADD, SIFT, PolyPhen-2, and Mutation taster), and clinical databases (ClinVar, OMIM, and dbSNP) along with manually curated database from the National Comprehensive Cancer Network (NCCN), the US Food and Drug Administration (FDA), CIVIC, Precision Cancer Therapy-MD Anderson, OncoKB, Pharmacogenomics Knowledgebase (PharmGKB), and Clinical Trials and directed literature searches (19–36).

### 2.5 Bioinformatics analysis for tumor mutational signature

TMB is emerging as one of the potential biomarkers that help in predicting the response to Immune Checkpoint Inhibitor (ICI) therapy response. WES is considered to be the gold standard approach to arrive at the Tumor mutational burden burden in any cancer, and the same has been recommended by the Food and Drug Administration (FDA) in the approval of KEYTRUDA (pembrolizumab). A sequential three-level filtering approach was adapted from Parikh et al. (2020) for the removal of germline variants and retention of possible somatic mutation. In level 1 filtering (tolerant approach), global-population frequency and South-Asian frequency were used to remove the polymorphic variants (> 1% of the population) (5, 37) from the cohort. In level 2 filtering (stringent approach), the variant allele frequency was used to remove the germline heterozygous variants. In level 3 filtering (baseline approach), variants were filtered based on the in-house baseline (germline samples). The baseline (reference genome pattern) was created by pooling together the blood samples of healthy individuals. With the use of this algorithm, following a robust machine learning approach, the TMB pipeline was throughly verified with tumor-normal and tumor-only samples to derive the whole exome TMB pattern (38). 

The microsatellite status was determined using a machine learning (ML)-based tool MSI-sensor2 (34). MSI occurs when mismatch repair (MMR) proteins are mutated, resulting in the accumulation of errors. The MSI status (based on the number of repeats) can be determined by polymerase chain reaction (PCR) on MMR immunohistochemistry (IHC) or by using the NGS technique. The presence of MSI-H in any tumor indicates a defect in the DNA repair mechanism. Based on our internal validation, the cutoff for high-TMB was considered 20 mutation/Mb, while patients with ≥15% MSI score were considered MSI-high (unpublished 4baseCare data).

### 2.6 Clinical reporting

Clinically relevant variants were prioritized and correlated to identify the markers associated with therapeutic response, prognosis, or supporting diagnostic evidence for any given patient’s tumor. These variants were interpreted and summarized in the report as per the American College of Medical Genetics and Genomics/American Society of Clinical Oncology/Association for Molecular Pathology/College of American Pathologists/European Society for Medical Oncology (ACMG/ASCO/AMP/CAP/ESMO) and NCCN guidelines (39, 40).

## 3 Results

### 3.1 Analytical validation of genomic alterations —accuracy, sensitivity, and limit of detection

To estimate the analytical accuracy, the limit of detection, and sensitivity for small variant calling (SNVs/INDELS), we used the National Institute of Standards and Technology (NIST) traceable reference standard from Horizon Discovery HD832 and cancer cell lines (obtained from the National Centre for Cell Sciences (NCCS), Pune). The variant and the variant allele frequencies (VAFs) detected were compared to the known VAFs given by the manufacturer/reference standard. **Table S2** summarizes results from Horizon reference standard HD832 demonstrating 100% concordance. Although genomic alterations below 5% were detected through the standard somatic pipeline from DRAGEN, at 1.9%, 2.8%, and 0.9%, the support of the mutant reads was less than 10. There was 98.7% concordance from the cell line data (**Table S3**). Similarly, gene fusion also showed 100% concordance using the Horizon reference standard (targeted FFPE RNA fusion reference standard; HD784-ST7) and 10 publicly available data from the Sequence Read Archive (SRA) database, which showed 100% concordance.

In our validation, when we generated libraries for HD832 using 200 ng as input DNA, we noticed that at 100X, we were unable to pick up any mutant rates that could confidently call for an *EGFR* exon 19 deletion. However, as we increased the depth of sequencing to 200X, we were able to pick up mutant reads correlating to exon 19 deletion as listed by Horizon Discovery. This enabled us to decide the minimum depth of coverage required to detect the INDELS in any given sample. We noticed that as we go lower in terms of the percentage of INDELS below 10%, allele frequency correlation or the mutant allelic burden correlation with high concordance was not achievable with adequate mutant read support, and hence, we had to limit our sensitivity/limit of detection (LOD) to 10% for INDELS. This could be due to the intrinsic limitation of the technology or the intrinsic nature of the reference/clinical sample or any other factor that could not be measurable or quantifiable. Hence, we have created a threshold for calling a variant a true variant provided that the variant has at least 10 good-quality mutant reads supporting the alternate read depth. The same threshold has been used throughout the analysis for all the patient data, reference material, and proficiency testing samples for any exonic deletions.

In our validation, the scope for detection of deletions is observed to be <20 bps. The sensitivity of detection of deletions from FFPE DNA depends to a large extent on the quality of the input DNA provided; if the input DNA is heavily fragmented, then the PCR amplifiability is affected and hence the library preparation.

The gene fusions are prioritized based on the confidence score provided by the DRAGEN workflow. Those gene fusions, which are true, have their scores listed with high confidence and minimum read support as five. Subsequently, as part of our in-house pipeline, the fusion reads are clinically annotated using public domain fusion databases such as FusionGDB and ChimeraDB. The well-studied functional fusion with diagnostic/prognostic/predictive biomarkers is further considered for clinical reporting. Those gene fusions, where the open reading frame (ORF) is lost, are not considered for further analysis.

### 3.2 Analytical validation of mutational signatures

**Table S3** demonstrates analytical accuracy for MSI and TMB calculation using an NGS- based approach on whole exome data. Six cancer cell lines (C33A, DU145, HCT-15, HCT-116, Jurkat6, and MOLT-4) with MSI-high status were found to have >22% MSI score (≥15% MSI score is considered as MSI-High), which corroborates with literature evidence (41–46). In addition to this, cell lines with high-MSI scores were also found to have TMB scores of >18 mutations/Mb. The T-47D breast cancer cell line was found to have a 7.58% MSI score (MSI-low or microsatellite stable (MSS)) and a TMB score of 3 mutation/Mb (TMB-low), which aligns with the literature evidence (46). Although the number of cancer cell lines used in this validation, with known MMR/MSI status, was less/limited, we have attempted to cover a wide range of primary tumor sources.

### 3.3 Clinical validation of tumor genomic alterations

The clinical validation of the WES (DNA and RNA)- based assay was established by comparing the result of the 106 assays with the corresponding conventional orthogonal methods [IHC, real-time PCR, Sanger sequencing, and fluorescence *in situ* hybridization (FISH)] and targeted NGS panels. This study of 62 SNVs/INDELS (biomarkers: *EGFR*, *KRAS*, *BRAF*, *KIT*, *PDGFRA*, *MLH1*, *MSH2*, *MSH6*, *PMS2*, *ATM*, *BRCA1*, *BRCA2*, *FANCA*, *CHEK1*, *ATR*, *PALB2*, *PBRM1*, *POLE*, *POLD1*, *PTEN*, *B2M*, *JAK1*, *JAK2*, *MDM2*, *MDM4*, and *DNMT3A*); 16 CNAs (biomarkers: *ERBB2*, *MET*, *CDK4*, *MDM2*, *BCL2L1*, and *MCL1* amplification) and 10 gene fusions (*ALK*, *ROS*, *RET*, and *NTRK* fusions) depicted 100% concordance when compared to conventional testing and targeted NGS panels (**Table 1** and **Tables S4**-**S6**).

**Table 1:** Clinical validation for genomic alterations using WES (DNA and RNA)- based assay.

Variant category	Validation sample	Source of samples	Biomarkers	Orthogonal test	WES Results	References
**SNVs/INDELS**	62 samples	FFPE or fresh frozen tissue and blood samples	*EGFR*, *KRAS*, *BRAF*, *KIT*, *PDGFRA*, *MLH1*, *MSH2*, *MSH6*, *PMS2*, *ATM*, *BRCA1*, *BRCA2*, *FANCA*, *CHEK1*, *ATR*, *PALB2*, *PBRM1*, *POLE*, *POLD1*, *PTEN*, *B2M*, *JAK1*, *JAK2*, *MDM2*, *MDM4*, and *DNMT3A*	Conventional RT-PCR test, IHC test, Sanger along with interlab comparison by targeted NGS	100% concordance	(47–49)
**Copy Number Alteration (Cutoff ≥ 6)**	16 samples	FFPE	*ERBB2*, *MET*, *CDK4*, *MDM2*, *BCL2L1*, and *MCL1* amplification	Conventional FISH test, IHC test along with interlab comparison by targeted NGS	100% concordance	(50–54)
**Gene Fusion** **(minimum of 5 unique read support spanning the junction boundaries)**	20 samples	FFPE	*ALK*, *ROS*, *RET*, and *NTRK* fusions	Conventional FISH test, IHC test along with interlab comparison by targeted NGS	100% concordance	(55–58)
**Microsatellite Instability (MSI)**	8 samples	FFPE	Microsatellite loci across the genome	Conventional *IHC* test on *MLH1*, *MSH2*, *MSH6* and *PMS*	100% concordance	(59, 60)

WES, whole exome sequencing; SNVs, single-nucleotide variants; INDELS, insertions/deletions; FFPE, formalin-fixed paraffin-embedded; IHC, immunohistochemistry; NGS, next-generation sequencing; FISH, fluorescence in situ hybridization.

### 3.4 Clinical validation of mutational signatures

**Figure 4:**
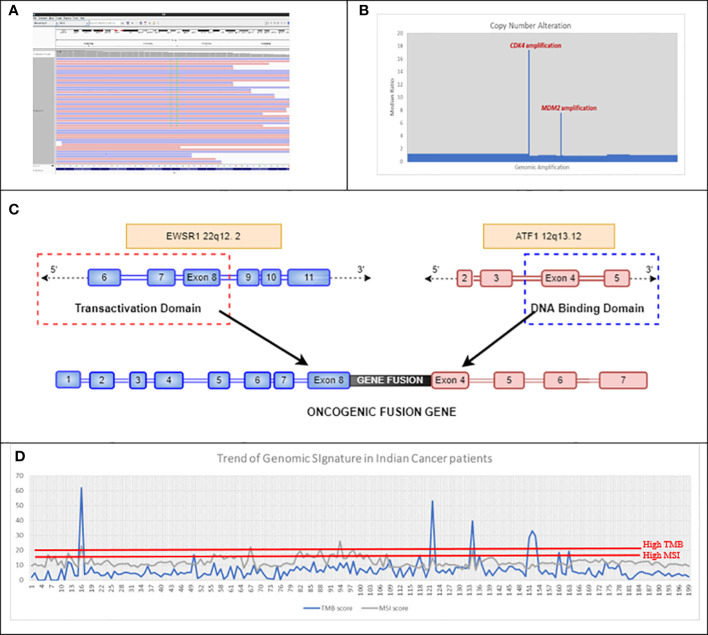
**(A)** Double mutation detected in exon 12 of *KRAS*, which led to an MNV change of *KRAS* G12F in Case 4BC-19. **(B)** Representative image of CNA detected using in-house bioinformatics pipeline, which showed amplification of *CDK4* and *MDM2* genes in Case number 4BC-63. **(C)** Schematic of the *EWSR1*::*ATF1* fusion gene detected in case number 4BC-146. The *EWSR1*::*ATF1* rearrangement is caused by the fusion of the N-terminal transactivation domain of *EWSR1* gene with the C-terminal DNA binding domain of *ATF1* gene. **(D)** Trend of mutational signatures (TMB and MSI) in 200 Indian cancer patients (unpublished 4baseCare data). The cutoff for MSI-H is >15 and that for TMB-H is >20. MNV, Muti-Nucleotide Variant; CNA, Copy Number Alteration; TMB, Tumor Mutational Burden; MSI, Microsatellite Instability.

We compared MSI calculation from eight clinical samples (using an in-house approach on exome data) with results from conventional IHC tests on four genes, namely, *MLH1*, *MSH2*, *MSH6*, and *PMS2*, which are used to detect the status of MMR in these patients. Our results showed 100% concordance with the results of IHC (**Table S7**). Moreover, our NGS assay focuses on loci of multiple microsatellite regions throughout the genome of the tumor cells, rather than selected short tandem repeats. The landscape of the mutational signatures depicted that TMB and MSI showed similar trends as detected by NGS in cancer patients’ cohort [unpublished 4baseCare data from 200 patients; the percentage of patients with high TMB was approximately 14% and that of high MSI was approximately 3% (**Figure 4D**)]. Identification of mutational signatures in sporadic cancer patients was useful in establishing prognosis and was predictive of response to certain chemotherapeutic regimens as well as immunotherapy.

### 3.5 Clinical reproducibility for the complete scope of analysis

Reproducibility was measured by sequencing Horizon libraries along with four independent clinical libraries. In line with requirements for clinical validation, we here demonstrated the reproducibility using horizon reference standard and along with four independent clinical samples to detect the SNVs (5% LOD), INDELS (10% LOD), and gene fusions. The experiments included 20 SNVs/INDELS and five gene fusions, which were reproduced in all the libraries that depict 100% reproducibility of the assay (**Table S8**).

### 3.6 Inter-lab comparison

As part of external quality assurance, 18 samples (**Table S11**) from proficiency testing programs for different cancer types (breast, lung, pancreatic, prostate, and ovarian) demonstrated 100% concordance using an in-house pipeline (unpublished 4baseCare data). In addition, inter-laboratory comparison with other NGS panels for 10 clinical samples showed 100% concordance in results (**Tables S4**, **S7**).

### 3.7 Genotype–phenotype correlation for hereditary and sporadic cancers

Four families (10 individuals) with a family history of hereditary cancer syndromes had undergone WES DNA blood based assay along with Sanger sequencing (4BC-113 to 4BC-122). Both the test results revealed the presence of pathogenic *BRCA1* frameshift mutation in two families and a pathogenic missense variant in *RAD50* gene in the third family, which is associated with hereditary breast and ovarian cancer (HBOC) syndrome. The fourth family had *STK11* pathogenic mutation, which is associated with the Peutz–Jeghers syndrome (**Table S10** and **Figure S3**). In addition to this genotype–phenotype correlation in hereditary cancer syndrome, we also identified pathogenic mutations (SNVs/INDELS and gene fusions) in seven patients with sporadic cancers that correlated with the clinical presentation (**Table S9**). In sporadic cancers, this extensive approach of CGP facilitates the discovery of rare mechanisms of resistance in different cancers such as hormone-positive breast cancer, non-small cell lung cancer, and castration-resistant prostate cancer in disease progression, that could help in prognostication as well as changing the treatment modality. We also identified some of the rare driver mutations in non-small cell lung cancer that could explain how genomics could help in predicting the treatment efficacy/inefficacy.

## 4 Discussion

The advantages of the WES approach for tumor DNA and RNA, over targeted NGS panel or RT-PCR, includes the identification of the broad spectrum of mutations (both driver and passenger) that helps in understanding tumor complexity and hence provides a comprehensive picture of the disease biology. As an outcome of this validation, we could detect the classical actionable driver mutations [*EGFR* exon 19 deletions in non-small cell lung cancer (NSCLC) patients (**Figure S4**)] and complex mutations and gene fusions. A few interesting case examples are described below from the validation cohort.

NGS as a technology stands out as more accurate for clinical management, as it can detect rare/complex muti-nucleotide variants (MNVs) that could be missed by conventional RT-PCR/genotyping techniques (predetermined assays). *KRAS* codon 12 (G12C) is one of the recently approved biomarkers for targeted therapy in lung cancer. The approval for sotorasib was based on CodeBreaK 100, a multicenter, single-arm, open-label clinical trial (NCT03600883) that included patients with locally advanced or metastatic NSCLC with *KRAS* G12C mutations. The overall response rate (ORR) observed in patients with *KRAS* G12C-mutated NSCLC treated with sotorasib (n = 124) was 36% [95% confidence interval (CI), 28–45]. In our study (case 4BC-19), real-time PCR of a rectal adenocarcinoma patient showed the expression of two different clonal mutations (G12V and G12C) at the same codon position (codon 12) of *KRAS* gene (**Figure 4A**). However, NGS revealed the presence of two neighboring nucleotides from the same codon 12 being mutated, thus depicting a complex event, resulting in an amino acid change from glycine to phenylalanine (G12F). This identification of complex nucleotide substitution and subsequent change in an amino acid is crucial in order to make treatment-related decisions for targeted therapy options.

Gene fusions in *FGFR2, FGFR3, NTRK1, NTRK2, NTRK3*, *ROS1*, *ALK*, and *RET* are frequently observed in NSCLC. However, case 4BC-146 revealed the presence of rare fusions of *EWSR1::ATF1* in NSCLC patient (**Figure 4C**). In this case, the outcome from the NGS is more toward understanding the molecular drivers associated with disease onset and its phenotype, since *EWSR1* fusions do not have targeted therapy drugs approved (47). These data elucidate the importance of RNA exome sequencing to identify rare/novel driver mutations associated with the disease (59). Such an exercise could also provide leads for novel targeted therapy as well as chemotherapy options.

A comprehensive NGS approach can also help in the identification of biomarkers associated with disease progression as depicted in case 4BC-63 with liposarcoma that revealed *CDK4* and *MDM2* amplification (**Figure 4B**). This corroborates the study by Ricciotti et al., where the authors showed that high levels of *MDM2* and *CDK4* amplification were associated with decreased disease-free survival (DFS) (p = 0.0168 and p = 0.0169, respectively) and disease-specific survival (DSS) (p = 0.0082 and p = 0.0140, respectively) (60).

In some patients whose tumor is defined by complex histologies, and or with rare tumor types, where there are no standard of care guidelines for clinical management, tailor-made targeted therapy based on the findings from comprehensive genomic profiling of the patient’s tumor is being explored as options in a single subject clinical trial setting (48). In addition to genomic alteration status, WES remains a gold standard for the derivation of mutational signatures, particularly, TMB and MSI, which are known immunotherapy biomarkers. The correlation between TMB and MSI is highly debatable. The majority of MSI-high (MSI-H) tumors happened to be TMB-high (TMB-H); however, not all TMB-H tumors are essentially MSI-H (52). In this study, we observed that MSI-H cell lines were found to have TMB scores on the higher side.

We have used DRAGEN Bio-IT™ platform, which is faster than the GATK with the BWA-MEM2 pipeline earlier established by Broad Institute. DRAGEN uses a type of hardware called field-programmable gate arrays (FPGAs) to deliver phenomenal speed-ups to their GATK-based short (SNV/INDELS) variant discovery pipeline (53). The time taken for exome analysis is less than 30 min on DRAGEN as compared to a few hours on the GATK (BWA-MEM2) workflow (61); also, DRAGEN has an advantage of improved sensitivity and specificity over the latter.

As part of the validation beyond the conversion of FASTQ to VCF using DRAGEN, we developed a pipeline for variant annotation, MSI, and TMB calculation along with curation for gene fusion. Most of the algorithms and database source here were publicly available. However, as per the CAP-AMP recommendation, any laboratory-developed test, or RUO NGS panel require analytical and clinical validation before it gets implemented for clinical use. Our validation study demonstrated here is one such effort.

In brief, WES (DNA and RNA)- based assay is a comprehensive NGS panel that targets approximately 22,000 unique genes to detect tumor genomic alterations. The assay is well validated with 97.5% specificity, 100% sensitivity, 98.2% accuracy, and 100% reproducibility to detect low-frequency somatic variants (**Table S12**). The customized in-house bioinformatics pipeline and data curation along with Illumina DRAGEN workflow for detection, annotation, and classification of genomic alterations provide a complete end-to-end workflow. The clinical report summarizes the genomic alterations as per the standard recommendations.

Together, this study summarizes a robust NGS testing that could provide the most comprehensive genomic information from the tumor DNA and RNA, describing the potential application of CGP in the clinic and correlating the clinical phenotype with the molecular findings for better disease stratification and treatment decisions for individual cancer patients.

## Limitations

A major limitation of our validation process is that there is no globally available guideline for WGS/WES in the clinic. The infrastructure access, together with trained resources (with technical and scientific capabilities) for performing such a complex genome sequencing assay, is a limitation in many community hospitals or tertiary care settings. To bridge the gap and to bring this service available to each cancer patient, we would require a strong collaboration between the industry, academia, and the government to support such an effort. Also, the major interfering substances that would impact the assay performance includes: a) cold ischemic time b) tissue fixation time, c) buffer used for tissue fixation neutral buffered formalin (NBF) and its pH, d) quality of wax used, e) presence of necrosis, and f) decalcification due to the presence of bony component.

Although the cost of NGS for whole exome was a bottleneck in extending the application of this tool in the clinical setting for patients of all economic strata, major technology leaders such as Illumina have constantly been striving to bring down the cost of genome sequencing to support cancer care and management. Owing to their developments, WES has become an economically viable option in the last two years with the cost being less than $500/exome. Beyond the ability to decipher the complex tumor heterogeneity that is inherent in many cancers, the adaptation of exome sequencing in the routine clinics could help the clinical community in better management of rare cancers at presentation as well as when the patient reaches a roadblock after multiple lines of therapy as per the standard of care.

## Data availability statement

The datasets presented in this study can be found in online repositories. The name of the repository/repositories and accession number(s) can be found in the article/**Supplementary Material**.

## Ethics statement

Informed consent was obtained from the enrolled patients for the use of de-identified data for research publications. The study was conducted according to the principles of the Declaration of Helsinki and as per the ICH and GCP guidelines. The study was approved by an independent ethics committee and review board (JCDC, India). The patients/participants provided their written informed consent to participate in this study.

## Author contributions

Conception and design: VV. Provision of study material or patients: in-house walk-in patients at 4baseCare, HG and KR. Collection and assembly of data: VJ, NT, LV, AK, PS, NM, and NJ. Data analysis and interpretation: RM, VJ, MC, BS, NT, and VV. Manuscript writing: RM, VJ, MC, NT, KP, and VV. Final approval of manuscript: all authors. Accountable for all aspects of the work: VV and HG.
