# Supplementary material for: An absolute approach to using whole exome DNA and RNA workflow for cancer biomarker testing
Source: Front Oncol. 2023 Mar 13;13:1002792. doi: 10.3389/fonc.2023.1002792 (PMC10040847; doi:10.3389/fonc.2023.1002792)

**FIGURE S1**

Representative NGS library profile analysed by Tapestation 2000. A clear, distinct peak centered around 329bp can be seen. In the graph, X-axis shows library fragment length in base pairs and Y-axis depicts normalized arbitrary fluorescence units.

**
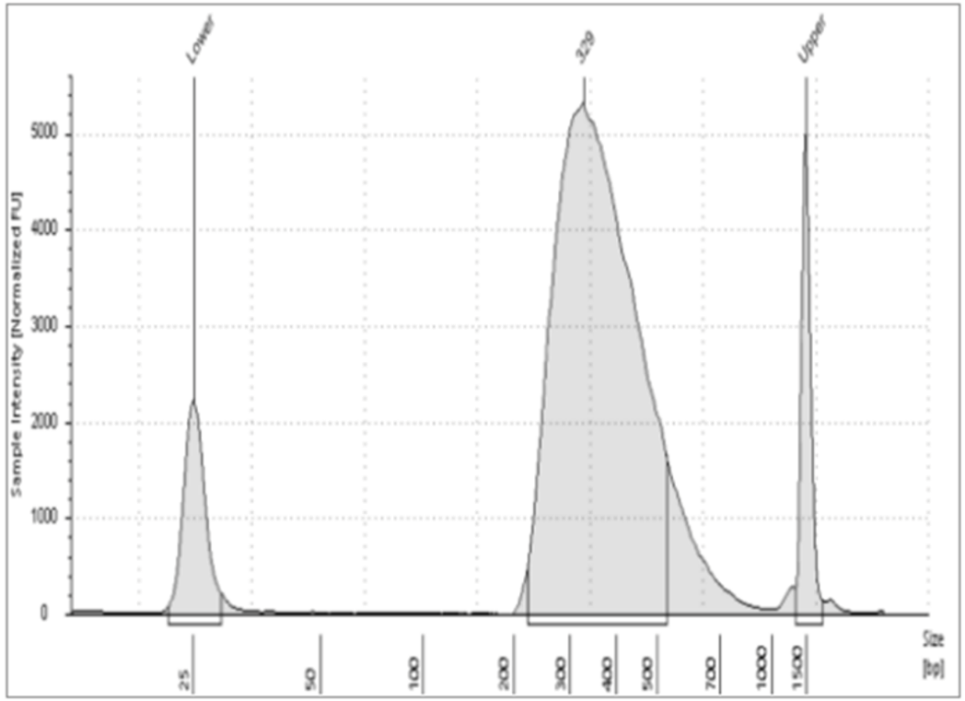
**

**FIGURE S2**

**(A)** Bioinformatic pipeline for DNA exome NGS data analysis (integrated with core DRAGEN workflow Somatic Pipeline. **(B)** Bioinformatic pipeline for RNA exome NGS data analysis (integrated with core DRAGEN workflow Somatic Pipeline

**
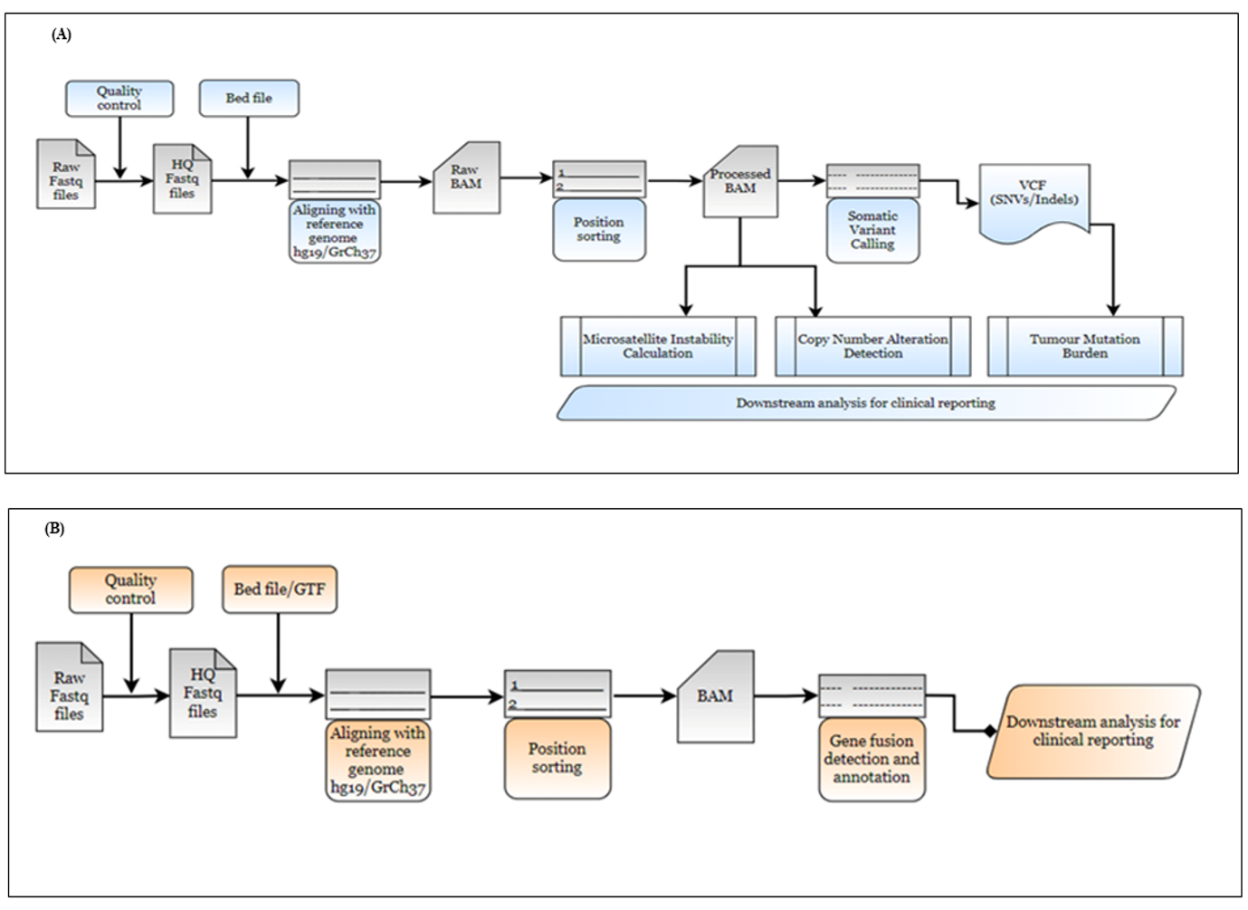
**

**FIGURE S3**

The pedigrees in the figure belong to four families with hereditary cancer syndromes. Pathogenic *BRCA1* frameshift mutation was detected in two families and a pathogenic missense variant in *RAD50* gene in the third family which is associated with HBOC syndrome. The fourth family had *STK11* pathogenic mutation which is associated with Peutz-Jeghers syndrome.

**
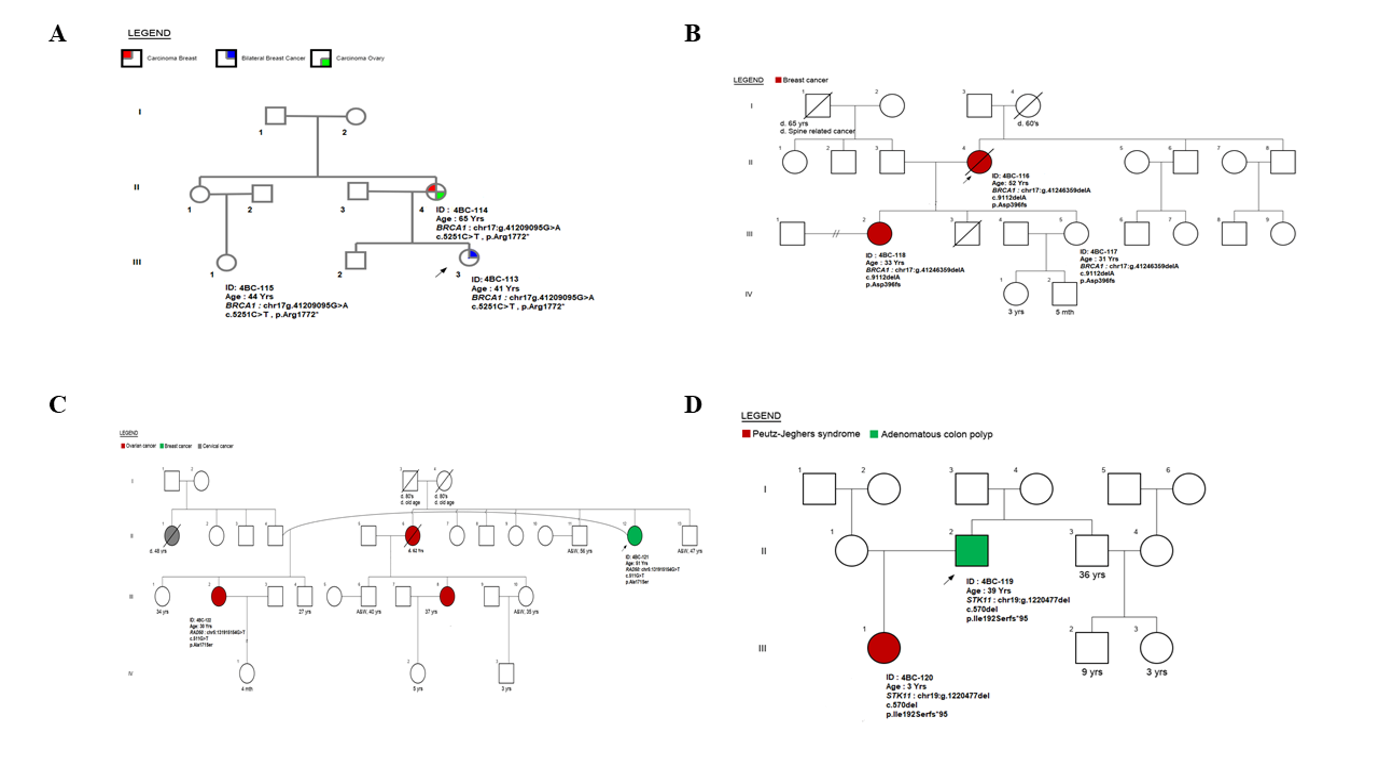
**

**FIGURE S4**

Representative image of the pathogenic in-frame deletion detected in exon 19 of *EGFR* gene in Case 4BC-10.

**
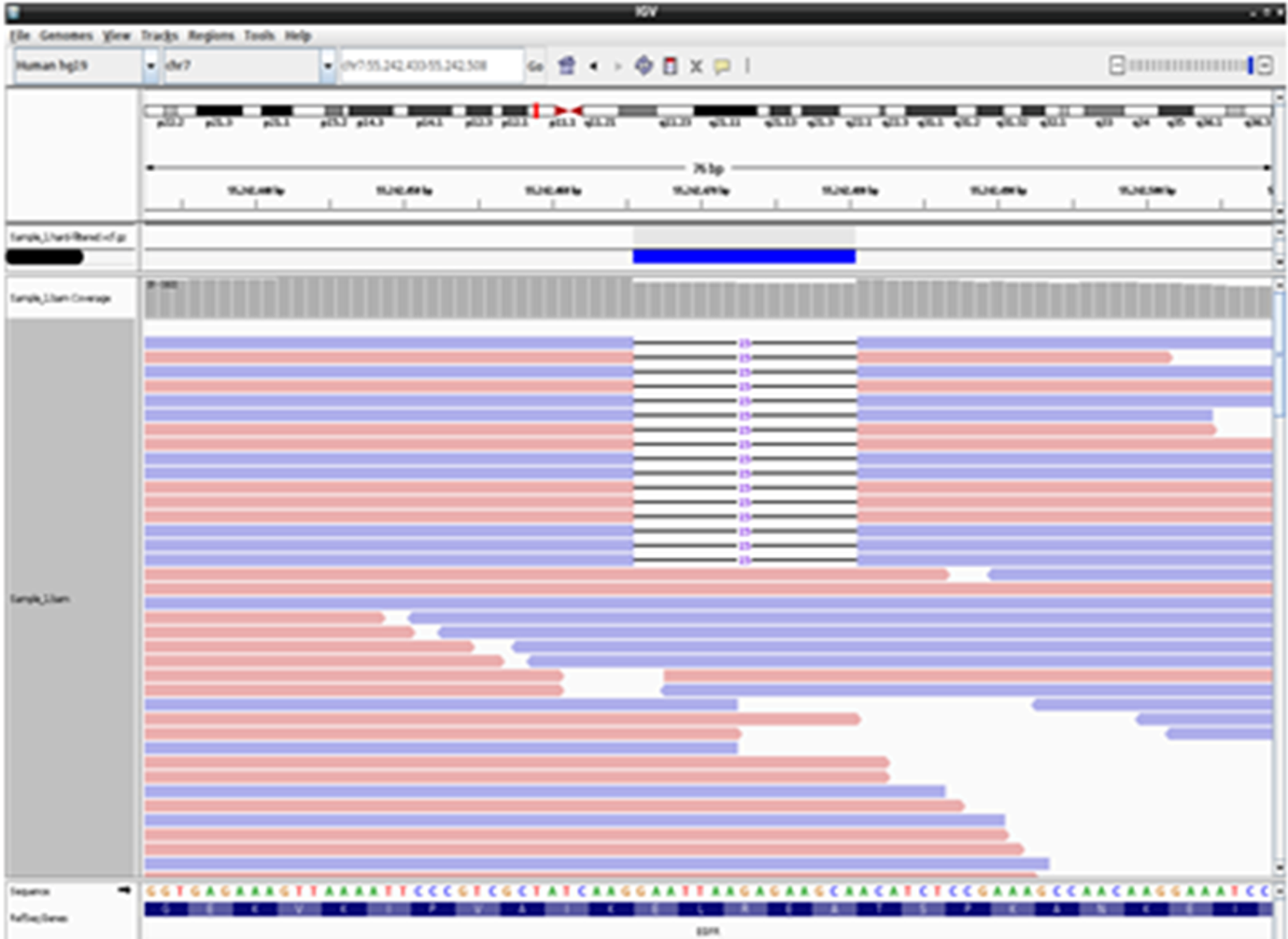
**

**FIGURE S5**

Variant Annotation and Prioritization: Variants from the raw vcf file are annotated based on multiple population databases and curated public-domain disease-specific databases: ClinVar, NCCN, FDA, CiViC, OncoKB, PharmGKB, and Precision Cancer Therapy-MD Anderson (Step 1). The variants are filtered based on the Phred score (>20) followed by the removal of non-coding variants. (Steps 2-3). Variant prioritized based on population frequency, disease databases, and clinical phenotype (Step 4).


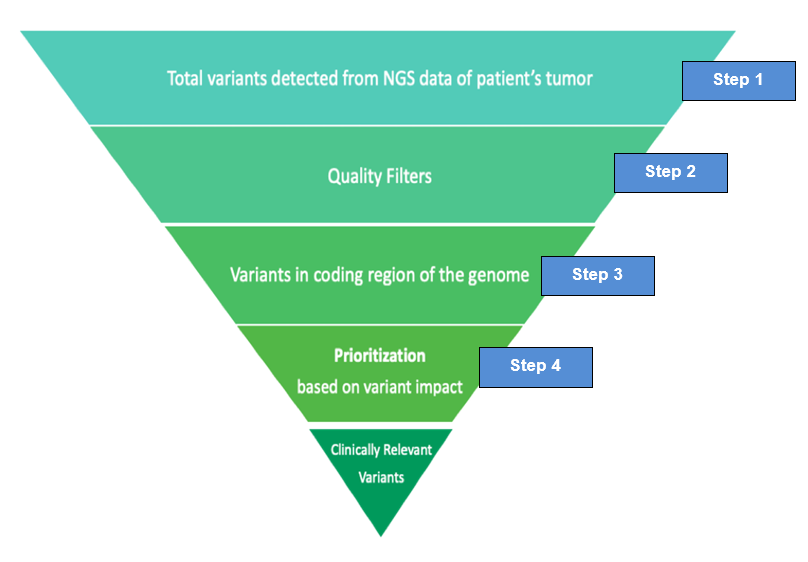

Supplement: Supplementary file 1 [file DataSheet_1.docx]
