# Supplementary material for: An absolute approach to using whole exome DNA and RNA workflow for cancer biomarker testing
Source: Front Oncol. 2023 Mar 13;13:1002792. doi: 10.3389/fonc.2023.1002792 (PMC10040847; doi:10.3389/fonc.2023.1002792)
Supplement: Supplementary file 2 [file Table_1.docx]

**Supplementary Tables**

**TABLE S1** Quality control parameters of WES (DNA&RNA) based assay

| Metric | Details |
| --- | --- |
| Tumor Content | >10% (>150 Tumor Cells/HPF) |
| Tumor Size | 5*5 mm^2^ |
| RNA Quantity | 125 to 500 ng input based on DV200 value |
| RNA Quality | ≥20% DV200 |
| DNA Quantity | 50-1000 ng input |
| DNA Quality | 260/280 ratio: 1.8-2.0 |
| DIN Value | >3 |
| Library Quality | ⁓300 bp (average size) |
| Library Quantity | ~10nM |
| Onboard Q30 | ≥90% |
| Depth of Coverage (DNA) | ≥ 200X |
| Uniformity of Coverage (DNA on target) | ≥90% at 50X |
| Total Reads (RNA) | ≥30 million |
| Percent Aligned (RNA on target) | ≥90% |

**TABLE S2** Analytical validation using Horizon standard for DNA (HD832) and RNA (HD784)

Note:

^b^Reproducibility after sequencing at different depths on consecutive days

^#^RS =Read support

- ^#1^ = In the case of reference Horizon standard RNA - HD784, the reference material provider, i.e., Horizon discovery provides us with qualitative results.

|  | Gene | Variant | Expected Variant Allele Frequency  (VAF) | Variant allelic frequency^b^ | | | Mean |
| --- | --- | --- | --- | --- | --- | --- | --- |
|  |  |  |  | **Day 1**  **(>500X)** | **Day 2**  **(~200X)** | **Day 3**  **(100X)** |  |
| Synthetic reference material from Horizon DNA (HD832) | *BRAF* | V600E | 13% | 10% | 8.6% | 10.5% | 9.7 |
|  | *KIT* | D816V | 10% | 9% | 8.7% | 9.9% | 9.2 |
|  | *EGFR* | ΔE746_A750 | 1.9% | 1.2% | 1.0% | Not Detected | - |
|  | *EGFR* | L858R | 2% | 2.72% | 5.36% | Not Detected | - |
|  | *EGFR* | T790M | 1.5% | 1.1% | 1.8% | Not Detected | - |
|  | *EGFR* | G719S | 23% | 20% | 15.2% | 20.5% | 18.57 |
|  | *KRAS* | G13D | 15% | 16% | 15.9% | 16.5% | 16.13 |
|  | *KRAS* | G12D | 6.3% | 5% | 5.3% | 5.8% | 5.37 |
|  | *NRAS* | Q61K | 11% | 12% | 10.2% | 12.6% | 11.6 |
|  | *PIK3CA* | H1047R | 16% | 17% | 14.8% | 16.2% | 16 |
|  | *PIK3CA* | E545K | 9.5% | 6% | 6.6% | 8.2% | 6.93 |
|  | *APC* | T1493T | 35% | 25% | 38% | 31.7% | 31.57 |
|  | *BRCA2* | K1691Nfs*15 | 33.5% | 31% | 25.5% | 31.8% | 29.43 |
|  | *CTNNB1* | S33Y | 37% | 31% | 27.3% | 30.1% | 29.47 |
|  | *CTNNB1* | S45del | 12% | 7% | 10.7% | 7.1% | 8.27 |
|  | *EGFR* | Q787Q | 15% | 16% | 8% | 16.7% | 13.57 |
|  | *FBXW7* | S668Vfs*39 | 35% | 33% | 29.4% | 33% | 31.8 |
|  | *MET* | A1357A | 7% | 7% | 7.4% | 7.7% | 7.37 |
|  | *MET* | L238fs*25 | 7% | 6% | 8.6% | 6.4% | 7 |
|  | *NOTCH1* | P668S | 29% | 25% | 23.6% | 25% | 24.53 |
|  | *TP53* | P72R | 92.5% | 92% | 92.5% | 92.4% | 92.3 |
| Synthetic reference material from Horizon RNA (HD784) | *SLC34A2::ROS1* | | - ^#1^ | ^#^RS:142 | RS:107 | RS:37 | 95.33 |
|  | *CCDC6::RET* | | - ^#1^ | RS:15 | RS:13 | RS:9 | 12.33 |
|  | *EML4::ALK* | | - ^#1^ | RS:14 | RS:13 | RS:6 | 11 |

| Cell line | Cell line source (ATCC) | Scientific evidence | | | WES Validation Results | | |
| --- | --- | --- | --- | --- | --- | --- | --- |
|  |  | **Reported Variants** | **MSI status** | **References** | **Variants detected** | **MSI score** | **TMB (Mut/Mb)** |
| C33A | Cervical Cancer | 6 | MSI-H | Seneviratne et al 2015^30^ | 6 | 24.58 | 18 |
| DU145 | Prostate Cancer | 7 | MSI-H | Panyutin et al 2017^31^ | 7 | 22.1 | 21 |
| HCT-15 | Colon Cancer | 16 | MSI-H | Gayet et al 2001^32^ | 15^a^ | 22.33 | 25 |
| HCT-116 | Colon Cancer | 10 | MSI-H | Gayet et al 2001^32^ | 10 | 28.23 | 20 |
| Jurkat6 | Leukemia | 5 | MSI-H | Brimmell et al 1998^33^ | 5 | 25.32 | 22 |
| MOLT-4 | Leukemia | 12 | MSI-H | Inoue et al 2000^34^ | 12 | 26.14 | 34 |
| T-47D | Breast Cancer | 2 | MSS | Lu et al 2013^35^ | 2 | 7.58 | 3 |

**TABLE S3** Analytical validation of SNV/INDELS, TMB and MSI status from cancer cell lines

Note: ^a^The single discordant variant could be due to passage differences

**TABLE S4** Clinical validation of SNVs and INDELS detected through WES (DNA&RNA) based assay

| Case No. | Cancer Type | Gene/Variant | Comparative analysis | | WES Validation Results |
| --- | --- | --- | --- | --- | --- |
|  |  |  | **Technique** | **Results** |  |
| 4BC-1 | Lung Cancer | *KRAS* G12S | Conventional RTPCR | Detected | Detected |
| 4BC-2 | Lung Cancer | *EGFR* negative | Conventional RTPCR | Not detected | Not detected |
| 4BC-3 | Lung Cancer | *EGFR* negative | Conventional RTPCR | Not detected | Not detected |
| 4BC-4 | Lung Cancer | *EGFR* negative | Conventional RTPCR | Not detected | Not detected |
| 4BC-5 | Lung Cancer | *EGFR* negative | Conventional RTPCR | Not detected | Not detected |
| 4BC-6 | Lung Cancer | *EGFR* negative | Conventional RTPCR | Not detected | Not detected |
| 4BC-7 | Lung Cancer | *EGFR* negative | Conventional RTPCR | Not detected | Not detected |
| 4BC-8 | Lung Cancer | *EGFR* negative | Conventional RTPCR | Not detected | Not detected |
| 4BC-9 | Lung Cancer | *EGFR* L58R | Conventional RTPCR | Detected | Detected |
| 4BC-10 | Lung Cancer | *EGFR* exon19 Del | Conventional RTPCR | Detected | Detected |
| 4BC-11 | Lung Cancer | *EGFR* L58R | Conventional RTPCR | Detected | Detected |
| 4BC-12 | Lung Cancer | *EGFR* exon19 Del | Conventional RTPCR | Detected | Detected |
| 4BC-13 | Lung Cancer | *EGFR* negative | Conventional RTPCR | Not detected | Not detected |
| 4BC-14 | Lung Cancer | *EGFR* negative | Conventional RTPCR | Not detected | Not detected |
| 4BC-15 | Non-Seminomatous Germ Cell Tumour | *EGFR* negative | Conventional RTPCR | Not detected | Not detected |
| 4BC-16 | Non-Seminomatous Germ Cell Tumour | *KRAS* G12S | Conventional RTPCR | Detected | Detected |
| 4BC-17 | Lung Cancer | *KRAS* G12D | Conventional RTPCR | Detected | Detected |
| 4BC-18 | Rectal carcinoma | *KRAS* G12D | Conventional RTPCR | Detected | Detected |
| 4BC-19 | Lung Cancer | *KRAS* G12F | Conventional RTPCR | Detected | Detected |
| 4BC-20 | Lung cancer | *BRAF* Wild Type | Targeted NGS panel | Not detected | Not detected |
| 4BC-21 | Melanoma | *BRAF* V600E | IHC test | Detected | Detected |
| 4BC-22 | GIST | *KIT* Wild Type | Sanger | Not detected | Not detected |
| 4BC-23 | GIST | *PDGFRA* Wild Type | Sanger | Not detected | Not detected |
| 4BC-24 | Lung cancer | *BRAF* Wild Type | IHC test | Not detected | Not detected |
| 4BC-25 | Lung cancer | *EGFR* Wild Type | RTPCR | Not detected | Not detected |
| 4BC-26 | Lung cancer | *KRAS* Wild Type | RTPCR | Not detected | Not detected |
| 4BC-27 | Lung cancer | *KRAS* Wild Type | NGS targeted panel | Not detected | Not detected |
| 4BC-28 | Lung cancer | *BRAF* Wild Type | NGS targeted panel | Not detected | Not detected |
| 4BC-29 | Lung cancer | *MLH1* Wild Type | NGS targeted panel | Not detected | Not detected |
| 4BC-30 | Lung cancer | *MSH2* Wild Type | NGS targeted panel | Not detected | Not detected |
| 4BC-31 | Lung cancer | *MSH6* Wild Type | NGS targeted panel | Not detected | Not detected |
| 4BC-32 | Lung cancer | *PMS2* Wild Type | NGS targeted panel | Not detected | Not detected |
| 4BC-33 | Lung cancer | *ATM* Wild Type | NGS targeted panel | Not detected | Not detected |
| 4BC-34 | Lung cancer | *BRCA1* Wild Type | NGS targeted panel | Not detected | Not detected |
| 4BC-35 | Lung cancer | *BRCA2* Wild Type | NGS targeted panel | Not detected | Not detected |
| 4BC-36 | Lung cancer | *FANCA* Wild Type | NGS targeted panel | Not detected | Not detected |
| 4BC-37 | Lung cancer | *CHEK1* Wild Type | NGS targeted panel | Not detected | Not detected |
| 4BC-38 | Lung cancer | *ATR* Wild Type | NGS targeted panel | Not detected | Not detected |
| 4BC-39 | Lung cancer | *PALB2* Wild Type | NGS targeted panel | Not detected | Not detected |
| 4BC-40 | Lung cancer | *PBRM1* Wild Type | NGS targeted panel | Not detected | Not detected |
| 4BC-41 | Lung cancer | *POLE* Wild Type | NGS targeted panel | Not detected | Not detected |
| 4BC-42 | Lung cancer | *POLD1* Wild Type | NGS targeted panel | Not detected | Not detected |
| 4BC-43 | Lung cancer | *PTEN* Wild Type | NGS targeted panel | Not detected | Not detected |
| 4BC-44 | Lung cancer | *B2M* Wild Type | NGS targeted panel | Not detected | Not detected |
| 4BC-45 | Lung cancer | *JAK1* Wild Type | NGS targeted panel | Not detected | Not detected |
| 4BC-46 | Lung cancer | *JAK2* Wild Type | NGS targeted panel | Not detected | Not detected |
| 4BC-47 | Lung cancer | *EGFR* Wild Type | NGS targeted panel | Not detected | Not detected |
| 4BC-48 | Lung cancer | *MDM2* Wild Type | NGS targeted panel | Not detected | Not detected |
| 4BC-49 | Lung cancer | *MDM4* Wild Type | NGS targeted panel | Not detected | Not detected |
| 4BC-50 | Lung cancer | *DNMT3A* Wild Type | NGS targeted panel | Not detected | Not detected |
| 4BC-51 | GIST | *KIT* Wild Type | Sanger | Not detected | Not detected |
| 4BC-52 | GIST | *PDGFRA* Wild Type | Sanger | Not detected | Not detected |
| 4BC-123 | Lung Cancer | *EGFR* exon19 Del | Interlab Comparison | Detected | Detected |
| 4BC-124 | Ovarian Carcinoma | *BRCA2* | Interlab Comparison | Detected | Detected |
| 4BC-125 | Lung Cancer | *KRAS* G12A, *MET* amplification | Interlab Comparison | Detected | Detected |
| 4BC-126 | Urothelial carcinoma (Bladder Cancer) | *TP53* R273L, *SDHC* and *MCL1, ASXL1, BCL2L1* amplification. | Interlab Comparison | Detected | Detected |
| 4BC-127 | Rectum carcinoma | *KRAS* G12C and *MSH2* Q593Ter | Interlab Comparison | Detected | Detected |
| 4BC-128 | Esophagus | *CDKN2A* P48R and *TP53* P190T | Interlab Comparison | Detected | Detected |
| 4BC-129 | Rectosigmaoid | *KRAS* G12C and *TP53* V272G | Interlab Comparison | Detected | Detected |
| 4BC-130 | Oral | *BRCA2* R2842C | Interlab Comparison | Detected | Detected |
| 4BC-131 | Ovary | *JAK2* V617F, *TP53* R282W and *ALK* D1203N | Interlab Comparison | Detected | Detected |
| 4BC-132 | Rectosigmaoid | *KRAS* G12D | Interlab Comparison | Detected | Detected |

**TABLE S5** Clinical validation of copy number alteration detected using WES (DNA&RNA) based assay

Note: Copy number >6 is considered as true amplification, while copy number >4 and <6 is considered equivocal, and <4 is negative for gene amplification.

| Sample ID | Cancer Type | Gene Name | Orthogonal Method | Result | NGS Result |
| --- | --- | --- | --- | --- | --- |
| 4BC-53 | Ca breast with liver metastasis | *ERBB2* Amplification | Conventional IHC | Detected | Detected |
| 4BC-54 | Carcinoma endometrium | *ERBB2* Amplification | Conventional IHC | Detected | Detected |
| 4BC-55 | Lung cancer | *ERBB2* Amplification | Other NGS methods | Not Detected | Not Detected |
| 4BC-56 | Lung cancer | *MET* Amplification | Other NGS methods | Not Detected | Not Detected |
| 4BC-57 | Ca stomach | *ERBB2* Amplification | Conventional IHC | Detected | Detected |
| 4BC-58 | Ca endometrium with liver metastasis | *ERBB2* Amplification | Conventional IHC | Detected | Detected |
| 4BC-59 | Rectum carcinoma | *ERBB2* Amplification | Conventional IHC | Detected | Detected |
| 4BC-60 | Breast cancer | *ERBB2* Amplification | Conventional IHC | Detected | Detected |
| 4BC-61 | Breast cancer | *ERBB2* Amplification | Conventional IHC | Detected | Detected |
| 4BC-62 | Ovarian cancer | *ERBB2* Amplification | Conventional IHC | Not Detected | Not Detected |
| 4BC-63 | Ca stomach (retroperitoneum) | *CDK4* Amplification & MDM2 | Targeted NGS panel | Detected | Detected |
| 4BC-64 | Bladder urothelial carcinoma | *BCL2L1* & *MCL1* amplification | Targeted NGS panel | Detected | Detected |
| 4BC-67 | Lung cancer | *MET* Wild Type | Targeted NGS panel | No amplification detected | Not detected |
| 4BC-68 | Lung cancer | *ERBB2* Wild Type | Targeted NGS panel | Not detected | Not detected |
| 4BC-65 | AML | *MET* amplification | Publicly Available Data, Downloaded from SRA Database | Known amplification | Detected |
| 4BC-66 | Breast cancer | *MET* amplification | Publicly Available Data, Downloaded from SRA Database | Known amplification | Detected |

**TABLE S6** Analytical and clinical validation of gene fusions

| Sample ID | Cancer Type | Sample source | Known fusion status from other targeted NGS methods | WES Validation Results |
| --- | --- | --- | --- | --- |
| 4BC-69 | Thyroid anaplastic | FFPE | *NTRK3::ETV6* Fusion positive | Detected (Read support 15) |
| 4BC-70 | Thyroid anaplastic | FFPE | *ETV6::NTRK3* Fusion positive | Detected (Read support 50) |
| 4BC-71 | Lung cancer | FFPE | *ROS1* Fusion negative | Not Detected |
| 4BC-72 | Lung cancer | FFPE | *ALK* Fusion negative | Not Detected |
| 4BC-73 | Lung cancer | FFPE | *ROS1* Fusion negative | Not Detected |
| 4BC-74 | Lung cancer | FFPE | *RET* Fusion negative | Not Detected |
| 4BC-75 | Lung cancer | FFPE | *ALK* Fusion negative | Not Detected |
| 4BC-76 | Lung cancer | FFPE | *ROS1* Fusion negative | Not Detected |
| 4BC-77 | Lung cancer | FFPE | *ALK* Fusion negative | Not Detected |
| 4BC-78 | Lung cancer | FFPE | *RET* Fusion negative | Not Detected |
| 4BC-85 | Lung cancer | FFPE | *EML4::ALK* Fusion positive | Detected (Read support 15) |
| 4BC-86 | Lung cancer | FFPE | *EML4::ALK* Fusion positive | Detected (Read support 8) |
| 4BC-87 | Lung cancer | FFPE | *EML4::ALK* Fusion positive | Detected (Read support 12) |
| 4BC-88 | CML | Cell line derived (fresh RNA) | *BCR::ABL1* Fusion positive | Detected (Read support 181) |
| 4BC-89 | CML | Cell line derived (fresh RNA) | *BCR::ABL1* Fusion positive | Detected (Read support 137) |
| 4BC-90 | CML | Cell line derived (fresh RNA) | *BCR::ABL1* Fusion positive | Detected(Read support 119) |
| 4BC-91 | AML | Cell line derived (fresh RNA) | *ETV6::NTRK3* Fusion positive | Detected(Read support 863) |
| 4BC-92 | AML | Cell line derived (fresh RNA) | *ETV6::NTRK3* Fusion positive | Detected (Read support 413) |
| 4BC-93 | AML | Cell line derived (fresh RNA) | *CBFB::MYH11* Fusion positive | Detected (Read support 28) |
| 4BC-94 | AML | Cell line derived (fresh RNA) | *CBFB::MYH11* Fusion positive | Detected (Read support 63) |

**TABLE S7** Clinical validation set for MSI calculation on WES (DNA&RNA) based assay

| Sample ID | Cancer Type | Comparative analysis | | WES Validation Results |
| --- | --- | --- | --- | --- |
|  |  | **Technique** | **Results** |  |
| 4BC-79 | Colon Cancer | Conventional IHC | MMR positive  (Loss of nuclear expression of *MLH1 & PMS2* proteins) | MSI-H  (MSI Score:19.8, VUS variant was detected in *MLH1* gene) |
| 4BC-80 | Rectum carcinoma | Conventional IHC | MMR positive  (Loss of nuclear expression of MMR proteins) | MSI-H  (MSI Score: 18.45, Pathogenic NULL variant was detected in *MSH2* gene) |
| 4BC-81 | Lung Cancer | Targeted NGS panel | MSS | MSS  (MSI Score:9.75, No clinically relevant variants were detected in MMR genes) |
| 4BC-82 | Lung Cancer | Targeted NGS panel | MSS | MSS  (MSI Score: 9.67, No clinically relevant variants were detected in MMR genes) |
| 4BC-83 | Colon Cancer | Conventional IHC | MMR positive  (Loss of nuclear expression of *MLH, MSH2* and *MSH6* proteins | MSI-H  (MSI Score: 38.22, Pathogenic variant was detected in *MSH2* gene) |
| 4BC-84 | UBC | Targeted NGS panel | MSS | MSS  (MSI Score:10.1, No clinically relevant variants were detected in MMR genes) |
| 4BC-165 | Rectal cancer with liver metastasis | Conventional IHC | MMR positive  (Loss of nuclear expression of *MSH2 & MSH6* proteins) | MSI-H  (MSI Score: 20.08, Pathogenic variant was detected in *MSH2* gene) |
| 4BC-166 | High Grade Endometrial Stromal Sarcoma | Conventional IHC | MMR positive  (Loss of nuclear expression of *MSH2 & MSH6* proteins) | MSI-H  (MSI Score: 19.04, Pathogenic variant was detected in *MSH2* & *MSH3* genes) |

**TABLE S8** Reproducibility in detection of genomic alterations from Horizon Reference Standards and clinical samples

| Sample ID | Source | Gene/Variant | Variant Type | Day 1 | Day 2 | Day3 |
| --- | --- | --- | --- | --- | --- | --- |
| 4BC-147 (Day 1), 4BC-148 (Day 2), 4BC-149 (Day 3) | FFPE | *TP53*  chr17:g.7579312dupC | SNV | ✓ | ✓ | ✓ |
| 4BC-150 (Day 1), 4BC-151 (Day 2), 4BC-152 (Day 3) | FFPE | *EGFR*  Chr7:g.55233024G>A | SNV | ✓ | ✓ | ✓ |
| 4BC-153 (Day 1), 4BC-154 (Day 2), 4BC-155 (Day 3) | Horizon DNA (HD832) | *BRAF, KIT , EGFR, KRAS, NRAS, PIK3CA, APC, BRCA2, CTNNB, FBXW7, MET, NOTCH1, TP53* | SNVs/  INDELS | ✓ | ✓ | ✓ |
| 4BC-156 (Day 1), 4BC-157 (Day 2), 4BC-158 (Day 3) | FFPE | *NTRK::ETV6* | Gene fusion | ✓ | ✓ | ✓ |
| 4BC-159 (Day 1), 4BC-160 (Day 2), 4BC-161 (Day 3) | FFPE | *NTRK::ETV6* | Gene fusion | ✓ | ✓ | ✓ |
| 4BC-162 (Day 1), 4BC-163 (Day 2), 4BC-164 (Day 3) | Horizon RNA (HD784) | *SLC34A2::ROS1,CCDC6::RET,EML4::ALK* | Gene fusions | ✓ | ✓ | ✓ |

**TABLE S9** Snapshot of genotype-phenotype correlation using CGP approach

| Sample ID | Clinical Presentation | Biochemical marker | NGS findings | Correlation |
| --- | --- | --- | --- | --- |
| 4BC-140 | A 59Y old female, hormone positive breast cancer treated the first line with endocrine therapy now presented with disease progression. Clinical suspicion of resistance to endocrine therapy. | ER+PR+Her2- | *ESR1::IMPG1*  (Exon 6 of *ESR1* and Exon 14 of *IMPG1*) | *ESR1* gene fusions is one of the known mechanisms of resistance to endocrine therapy. It represents 3-5% of all the *ESR1* alterations in breast cancer |
| 4BC-141 | 56Y old male with NSCLC diagnosed in 2019, *EGFR* positive on first line TKI, now presented with disease progression. *EGFR* T790M mutation was not detected. Clinical query to understand the mechanism of resistance. | Not applicable | *EGFR* (*EGFR* gain, *MET* amplification (copy number 12), *EGFR* (L858R, *TP53* delT, p.Asn288IlefsTer57) | *MET* amplification suggestive of resistance to *EGFR* TKI |
| 4BC-142 | 61Y old female presented with carcinoma right breast and a detailed workup revealed TNBC. Family history indicates sister was affected by cancer. Tumor profiling was suggested to screen for HRR mutations for further management as well as any suspected germline predisposition. | ER-negative PR negative Her2 negative | *BRCA1* (*BRCA1* c.81-4_81-1delCTAG) | ***BRCA1* pathogenic mutation** was identified (both in tumor as well as in germline) that is known to predispose individuals with increased risk of breast and ovarian cancer (**here the syndromic presentation is classical**) |
| 4BC-143 | 87Y old male diagnosed with metastatic prostate cancer, presented with disease progression after treatment with ADT (androgen deprivation therapy). Clinical suspicion to understand the mechanism of resistance to ADT and CRPC. |  | *TMPRSS2:: ERG* (Exon 2 of *TMPRSS2* and Exon 5 of *ERG)*, *TP53* R209X, *RBI* R787X | ***TMPRSS2* fusions are known to be associated with poor prognosis** in prostate cancer (36) |
| 4BC-144 | 47Y old male presented with mucinous signet ring cell adenocarcinoma of rectum, based on histology possibility of microsatellite instability was suspected. NGS profiling was advised. | No *ERBB2* amplification/expression, *MSH2*–Loss of nuclear expression, *MSH6*–Loss of nuclear expression, *MLH1*-Loss of nuclear expression, *PMS2*-Loss of nuclear expression | *KRAS* (*KRAS* p.G12D, *MSH2* p.Q593X) | The histology, **NGS and IHC findings together confirm microsatellite instability status as high** in this patient. This could help in further clinical management |
| 4BC-145 | 48Y old male diagnosed with adenocarcinoma of ascending transverse colon, presented with *KRAS* mutation. Family history revealed mother was diagnosed with colon cancer at an age of 59Y. Clinical suspicion of Lynch syndrome to be ruled out and NGS was advised. | *MLH1*–Loss of nuclear expression, *PMS2*–Loss of nuclear expression, *MSH6*–Intact nuclear expression, *MSH2* –Intact nuclear expression | *KRAS* (*KRAS* G12D, *ATM* R447X, *ATM* R 451C, *TP53* R175C, *CDKN2A* P113L, *MLH1^#^* Q60H). **MSI score 19.8 (high)** | IHC and **NGS-based** **microsatellite instability status from whole exome data** confirm the MSI status of the patient’s tumor |
| 4BC-146 | 54Y old female was diagnosed with NSCLC and was on standard-of-care therapy for 1.5 years. Now presented with disease progression in 2021. No classical driver mutations were detected. | Not applicable | *EWSR1::ATF1* | NGS findings revealed the presence of a rare sarcoma fusion in this patient’s tumor. A case demonstrating unusual presentation of NSCLC with novel molecular driver in this cancer type |

Note: ^#^*MLH1* Q60H is classified as VUS variant as per the ACMG criteria. However, the variant was neither found in ExAC nor 1000Genome and is classified as deleterious by SIFT, probably damaging by PolyPhen, disease causing by Mutation Taster and deleterious by Condel.

**TABLE S10** Additional samples associated with genotype-phenotype analysis

| Case No. | Cancer Type | Gene/Variant | Comparative analysis | | WES Validation Results |
| --- | --- | --- | --- | --- | --- |
|  |  |  | **Technique** | **Results** |  |
| 4BC-113 | HBOC | *BRCA1* | Sanger | Detected | Detected |
| 4BC-114 | HBOC | *BRCA1* | Sanger | Detected | Detected |
| 4BC-115 | Predisposed to HBOC | *BRCA1* | Sanger | Detected | Detected |
| 4BC-116 | HBOC | *BRCA1* | Sanger | Detected | Detected |
| 4BC-117 | Predisposed to HBOC | *BRCA1* | Sanger | Detected | Detected |
| 4BC-118 | HBOC | *BRCA1* | Sanger | Detected | Detected |
| 4BC-119 | Peutz-Jeghers syndrome | *STK11* | Sanger | Detected | Detected |
| 4BC-120 | Predispose to Peutz-Jeghers syndrome | *STK11* | Sanger | Detected | Detected |
| 4BC-121 | HBOC | *RAD50* | Sanger | Detected | Detected |
| 4BC-122 | HBOC | *RAD50* | Sanger | Detected | Detected |

**TABLE S11:** Proficiency Testing (European Molecular Genetics Quality Network) Samples

| Sample ID | Cancer Type | Technique | Expected Variant | Observed Variant | Observed Percent Allelic frequency | Concordance |
| --- | --- | --- | --- | --- | --- | --- |
| 4BC-95 | Lung Cancer | Proficiency Testing | *KRAS* | *KRAS* c.34G>T,  p.(Gly12Cys) | 23% | 100% |
| 4BC-96 | Lung Cancer | Proficiency Testing | *EGFR* | *EGFR* c.2303_2311dup p.(Ser768_Asp770dup) | 26% | 100% |
| 4BC-97 | Lung Cancer | Proficiency Testing | *EGFR* | *EGFR* c.2155G>A p.(Gly719Ser) | 9% | 100% |
| 4BC-98 | Lung Cancer | Proficiency Testing | *KRAS* | *KRAS* c.175G>A  p.(Ala59Thr) | 20% | 100% |
| 4BC-99 | Lung Cancer | Proficiency Testing | Not Detected | Not Detected | - | 100% |
| 4BC-100 | Lung Cancer | Proficiency Testing | Not Detected | Not Detected | - | 100% |
| 4BC-101 | Lung Cancer | Proficiency Testing | Not Detected | Not Detected | - | 100% |
| 4BC-102 | Lung Cancer | Proficiency Testing | *BRAF* | *BRAF* c.1798_1799delinsAA p.(Val600Lys) | 33% | 100% |
| 4BC-103 | Lung Cancer | Proficiency Testing | *EGFR* | *EGFR* c.2573T>G p.(Leu858Arg) | 9% | 100% |
| 4BC-104 | Lung Cancer | Proficiency Testing | Not Detected | Not Detected | - | 100% |
| 4BC-105 | Prostate | Proficiency Testing | *BRCA2* | *BRCA2* c.7977-1G>C | 45% | 100% |
| 4BC-106 | Prostate | Proficiency Testing | Not Detected | Not Detected | - | 100% |
| 4BC-107 | Ovarian | Proficiency Testing | Not Detected | Not Detected | - | 100% |
| 4BC-108 | Ovarian | Proficiency Testing | *BRCA1* | *BRCA1* c.5075-2A>C | 36% | 100% |
| 4BC-109 | Ovarian | Proficiency Testing | *BRCA1* | *BRCA1* c.5558dup p.(Tyr1853Ter) | 52% | 100% |
| 4BC-110 | Ovarian Breast, Pancreatic | Proficiency Testing | *CHEK2* | *CHEK2* c.1263del p.(Ser422Valfs*15) | 50% | 100% |
| 4BC-111 | Ovarian Breast, Pancreatic | Proficiency Testing | Not Detected | Not Detected | - | 100% |
| 4BC-112 | Pancreatic, Prostate Cancer | Proficiency Testing | *BRCA2* | *BRCA2* c.5722_5723del p.(Leu1908fs)  *BRCA2* c.5722_5723del p.(Leu1908Argfs*2) | 50% | 100% |

**TABLE S12** Performance metrics of WES (DNA&RNA) based assay validation (61)

| Parameter | Formula | Analytical | Definition |
| --- | --- | --- | --- |
| Specificity (62) | TP/(TP+FN) | 97.5% | The ability to correctly identify samples with genomic alterations |
| Sensitivity | TN/(TN+FP) | 100% | The ability to correctly identify samples without genomic alterations |
| Accuracy | TP+TN/(TP+TN+FP+FN) | 98.2% | The genomic alterations are predicted exactly as expected (to standard/InterLab test) |
| Reproducibility | Not Applicable (qualitative measure) | 100% | Results obtained by an *in silico* and wet lab process are to be achieved again with a high degree of reliability when the process is repeated |
| Limit of Detection (LOD) | Not Applicable (assessed using sensitivity standard as mentioned above) | 5% for SNV and 10% for INDELS (<10 bp) | The limit of detection (LOD) is defined as the lowest concentration of an analyte in a sample that can be consistently detected with a stated probability |
| PPV | TP/(TP+FP) | 100% | The probability that following a positive test result, that sample will truly have those specific genomic alterations |
| NPV | TN/(FN+TN) | 94.3% | The probability that following a negative test result, that sample will truly not have those specific genomic alterations |

Note: Total 252 genomic alterations in 166 patients, cell lines and reference standard, total TP=157, total TN=66, total FP=0, total FN=4. TP = True positive, FP = False positive, FN = False negative, TN = True negative. PPV=Positive Predictive Value, NPV=Negative Predictive Value.
